# Supplementary material for: Detecting the ecological footprint of selection
Source: PLoS One. 2024 Jun 7;19(6):e0302794. doi: 10.1371/journal.pone.0302794 (PMC11161045; doi:10.1371/journal.pone.0302794)
Supplement: S1 Table — All MESS model parameters, their interpretations and range of possible values. Parameters indicated with an asterisk (*) are pseudo-parameters which are either emergent, compound, or randomly sampled from a distribution with parameters determined by other elements of the model. Parameters for the simulations where either uniformly (†) or loguniformly (°) drawn in the range referenced as tested range, when applicable. The chosen ranges are based on [29]. (DOCX) [file pone.0302794.s001.docx]

**Supporting information**

**S1 Table 1. MESS model parameters.** All MESS model parameters, their interpretations and range of possible values. Parameters indicated with an asterisk (*) are pseudo-parameters which are either emergent, compound, or randomly sampled from a distribution with parameters determined by other elements of the model. Parameters for the simulations where either uniformly (^†^) or loguniformly (°) drawn in the range referenced as tested range, when applicable. The chosen ranges are based on [29].

| **Categorical parameters** | | | |
| --- | --- | --- | --- |
| **Parameter** | | **Options** | **Tested range** |
| Community assembly model | | Neutral / Competition / Environmental filtering / Pairwise competition /  *β*-competition | All options ^†^ |
| *In situ* speciation model | | None / Point mutation / Random fission | Point mutation |
| Local community initial conditions | | Metacommunity sample / Monodominance | Monodominance |
| **Metacommunity component parameters** | | | |
| **Symbol** | **Meaning of parameter** | **Type and range** | **Tested range** |
| *J_M_* | Total number of individuals | Integer 1 | 5e5 |
| *S_M_* | Total number of species | Integer *>* 1 | 250 |
| *λ* | Per lineage birth rate (speciation) | Real *∈* [0*,∞*] | 2 |
| ɛ | Per lineage death rate (extinction) as proportion of *λ* | Real *∈* [0*,* 1] | 0.7 |
| *σ*^2^*M* | Trait evolution rate variance (Brownian motion) | Real *>* 0 | 2 |
| **Local community component parameters** | | | |
| **Symbol** | **Meaning of parameter** | **Type and range** | **Tested range** |
| *J* | Total number of individuals | Integer *>* 1 | [1000, 5000] ^†^ |
| *S* | Local species richness* | Integer *>* 1 | *Not applicable* |
| *ν* | Per capita per birth speciation rate | Real *∈* [0*,* 1] | [0.0005, 0.005] ° |
| *m* | Immigration rate from metacommunity (per step) | Real *∈* [0*,* 1] | [0.001, 0.01] ° |
| *σ*^2^ | Trait evolution rate variance (per speciation event)* | Real *>* 0 | *Not applicable* |
| *z_E_* | Optimal trait value in environment* | Real | *Not applicable* |
| *s_E_* | Strength of ecological filtering | Real *>* 0 | [0.01, 10] ° |
| Λ | Fraction of turnover equilibrium* | Real *∈* [0*,* 1] | [0*,* 1] ^†^ |
| *β_intra_* | Strength of intraspecific competition | Real *>* 0 | [0.01, 1] ° |
| *β_inter_* | Strength of interspecific competition | Real *>* 0 | [0.01, 1] ° |
| **Population genetics coalescence component parameters** | | | |
| **Symbol** | **Meaning of parameter** | **Type and range** | **Tested range** |
| *L* | Sequence length of simulated genomic region | Integer *>* 0 | 570 |
| *µ* | Mutation rate | Real *∈* [0*,* 1] | 2.2e-8 |
| *α* | Abundance/Ne scaling factor | Integer *>* 0 | [1000, 10000] ^†^ |

**S1 Table 1: MESS model parameters**

All MESS model parameters, their interpretations and range of possible values. Parameters indicated with an asterisk (*) are pseudo-parameters which are either emergent, compound, or randomly sampled from a distribution with parameters determined by other elements of the model.

Parameters for the simulations where either uniformly (^†^) or loguniformly (°) drawn in the range referenced as tested range, when applicable. The chosen ranges are based on [29].
