# Supplementary material for: Detecting the ecological footprint of selection
Source: PLoS One. 2024 Jun 7;19(6):e0302794. doi: 10.1371/journal.pone.0302794 (PMC11161045; doi:10.1371/journal.pone.0302794)
Supplement: S2 Table — Results are for 50 simulations of 200 generations on a single core. (DOCX) [file pone.0302794.s002.docx]

**Supporting information**

**S2 Algorithm optimisation**

A pairwise comparison of trait values costs more computation time than a single comparison to the mean. As expected, the simulations for the pairwise competition model were slower (around 4 times) than for the mean competition model. Yet, the computation time is a critical issue in a context of large numbers of simulation experiments required for classifier training, and this necessitated code optimization.

| **Model** | **Before optimization: [29] with pairwise competition added by this study** | **After optimization: this study** | **Ratio** |
| --- | --- | --- | --- |
| **Neutral** | 9 *±* 2 | 24 *±* 3 | 0.375 |
| **Mean competition** | 456 *±* 114 | 89 *±* 11 | 5.14 |
| **Pairwise competition** | 1944 *±* 258 | 323 *±* 40 | 6.02 |
| **Environmental filtering** | 80 *±* 18 | 70 *±* 10 | 1.14 |

**S2 Table 2: Comparison of the speed of the simulations for different version of the code** (mean value per run, *±* standard deviation, in seconds). Results are for 50 simulations of 200 generations on a single core.
