# Supplementary material for: Detecting the ecological footprint of selection
Source: PLoS One. 2024 Jun 7;19(6):e0302794. doi: 10.1371/journal.pone.0302794 (PMC11161045; doi:10.1371/journal.pone.0302794)
Supplement: S3 Table — (DOCX) [file pone.0302794.s003.docx]

| Model | Mean competition | Neutral | Filtering |
| --- | --- | --- | --- |
| Percentage of pairwise simulations assigned to the model | 48% | 28% | 24% |

**S4 Machine learning assessment**

**S4 Table 3:** Inference of 100 simulations run under the pairwise competition model classified by a classifier trained only with mean competition, neutral and environmental filtering simulations.
