# Supplementary figures and images for: Detecting the ecological footprint of selection
Source: PLoS One. 2024 Jun 7;19(6):e0302794. doi: 10.1371/journal.pone.0302794 (PMC11161045; doi:10.1371/journal.pone.0302794)

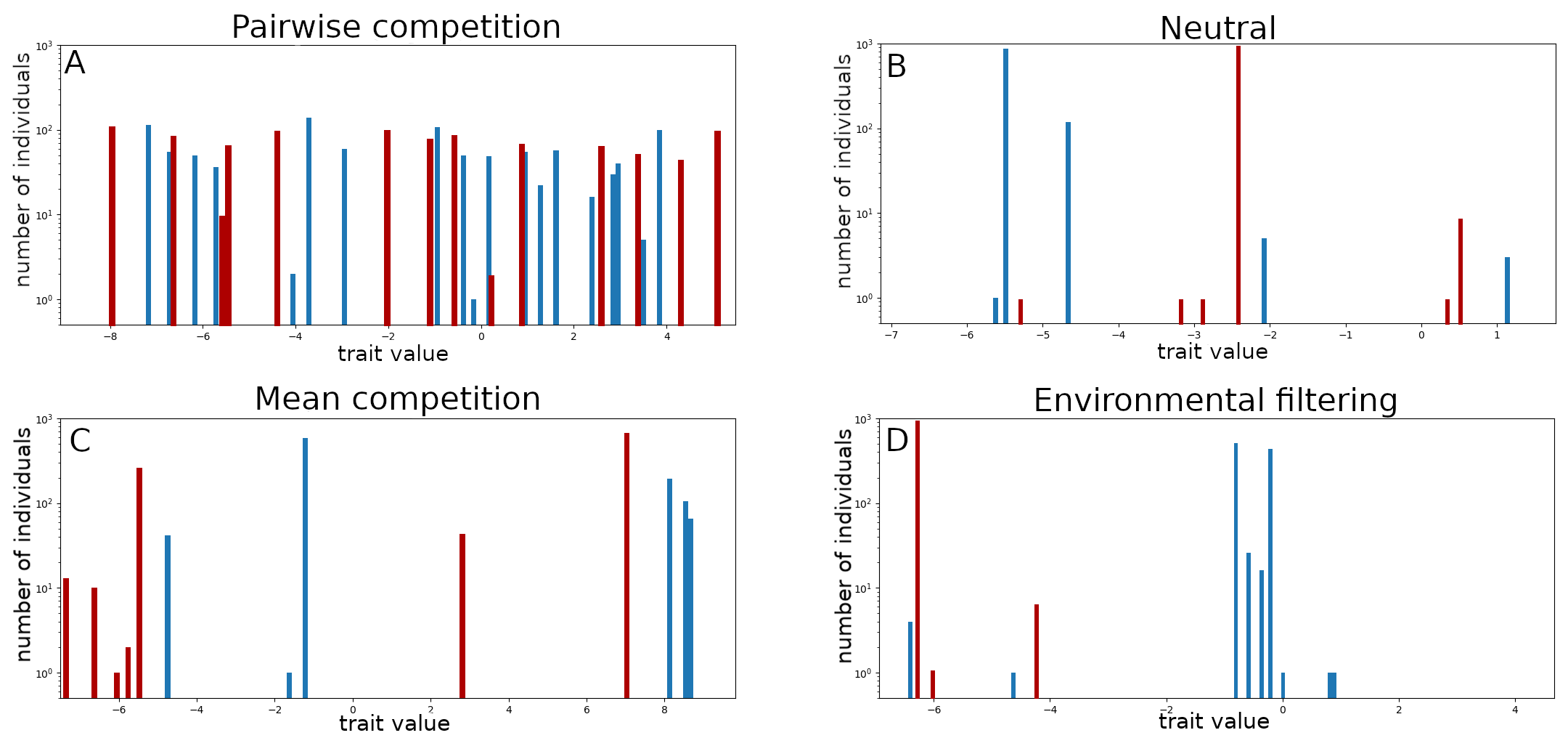

Supplement: S1 Fig — Two examples (red and blue) are given for each model. Two groups of species are distancing themselves in the mean competition model (C), while the species are much more grouped together in the environmental filtering case (D) and evenly distributed in the pairwise competition model (A). In the neutral case, they are random and their abundances follow a typical log-normal distribution. This also shows that we can expect significantly different results in the summary statistics resulting from trait data, but also in the species abundances and their variation and thus in the phylogeny. (TIF) [file pone.0302794.s004.tif]

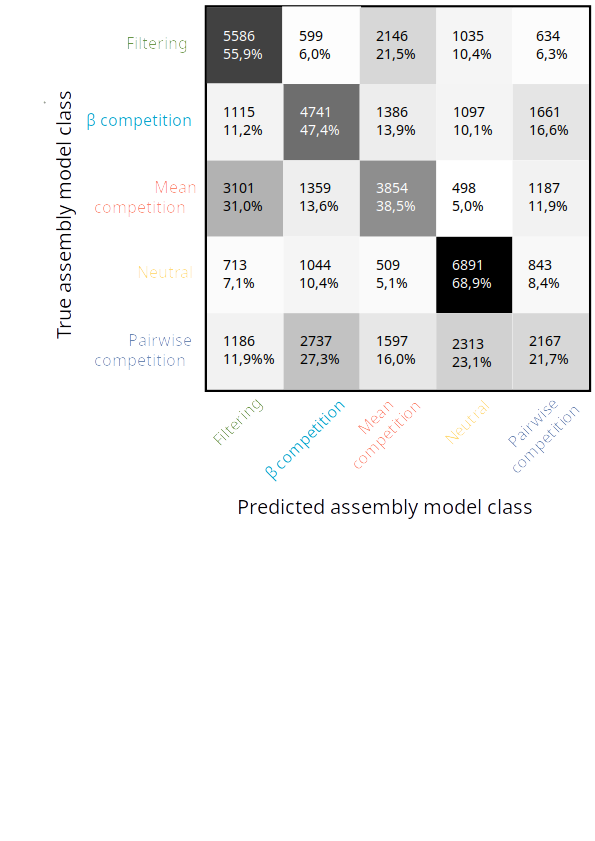

Supplement: S2 Fig — Percentages indicate the proportion of simulations run with one given class (raw) assigned to the column class. Mean competition is often mistaken for filtering, and pairwise competition for both neutrality and β-competition. (TIF) [file pone.0302794.s005.tif]
